# Meat Substitute Markets: A Comparative Analysis of Meat Analogs in Austria

**DOI:** 10.3390/foods12112211

**Published:** 2023-05-31

**Authors:** Christof Falkenberg, Alena Trexler, Christian Garaus, Siegfried Pöchtrager

**Affiliations:** Institute of Marketing & Innovation, Department of Economics and Social Sciences, University of Natural Resources and Life Sciences, Vienna, Feistmantelstrasse 4, A-1180 Vienna, Austria; alena.trexler@students.boku.ac.at (A.T.); christian.garaus@boku.ac.at (C.G.); siegfried.poechtrager@boku.ac.at (S.P.)

**Keywords:** innovative, meat alternative, market analysis, consumer, alternative protein, price, nutrient, ingredient, replication

## Abstract

The consumption of meat substitutes has significantly grown over the last decade. To understand the extent to which plant-based meat alternatives can already substitute conventional meat in terms of price and nutritional value, detailed knowledge of current market offerings is essential. We conducted an analysis of 38 plant-based minced products and 36 plant-based sausage products in Austrian supermarkets. The data were obtained using standardized observation in Austrian supermarkets reflecting 90% of the current market, expanded further through secondary data, and analyzed the generated dataset using mean value comparison. To provide a broader perspective on the trends in these markets, we incorporate results from a comparative study conducted in Australia. Our results obtained through *t*-tests revealed that there is no statistically significant difference in the protein content of plant-based meat substitutes and conventional meat (at the 95% confidence interval), underscoring the potential of meat substitutes as an alternative source of protein. Offering comparable protein content but with significantly lower caloric intake (at the 1% significance level), plant-based substitutes may contribute to reducing obesity in industrialized countries. The findings also reveal that plant-based products continue to be priced significantly higher than conventional meat (at the 1% significance level). We found substantial differences in ingredients and other nutritional values of plant-based products between Austria and Australia, although the main protein sources are the same in both countries, with peas being included in 60 out of 74 and soy in 27 out of 74 Austrian products. Our article concludes with a discussion of the implications for scholars and policymakers and identifies new avenues for future research.

## 1. Introduction

It is crucial to reduce global greenhouse gas emissions [1] and stay below the two degrees Celsius limit of the Paris Climate Agreement [2] to mitigate the current climate crisis and prevent more severe scenarios. According to Poore and Nemecek [3], food accounts for a quarter of the global carbon footprint. As meat is responsible for a large part of problematic greenhouse gases in food production [3,4], meat alternatives can be a good option to replace meat and thus contribute to climate protection [5]. The high land consumption associated with animal husbandry and feed production is also problematic from an environmental perspective. Almost half of the world’s habitable land is used for agriculture, of which 77% is used as grazing land for animal feed or feed production [6]. This means that only a small proportion of the land suitable for agriculture is currently used directly for growing crops for human consumption.

From a societal point of view, the continued increase in meat consumption worldwide raises both ethical and health concerns. Since 1960, meat production worldwide has increased fivefold [7]. Global meat consumption is still growing [8], although high meat consumption is associated with severe health risks such as more cases of type two diabetes, colorectal cancer, coronary heart disease, and other forms of cardiovascular and zoonotic diseases [9,10,11]. Data from Statistics Austria and AMA Marketing [12] show that Austrians ate an average of 58.9 kg of meat per capita in 2021. This is about three times the amount recommended by the Austrian Ministry of Social Affairs [13]. Meat alternatives offer versatile solutions to these aforementioned problems. Plant-based meat alternatives, in particular, are already readily available to the average person in Austria and are currently the most commonly consumed alternative to meat [14].

More and more people are considering meat alternatives for a variety of reasons (e.g., climate crisis, health, and animal welfare) [15,16,17]. In the absence of global regulations for the naming of these products, there is a concern that consumers may be led to believe that plant-based meat alternatives have similar nutritional compositions to their meat equivalents because of their names [18]. Yet according to Kumar et al. [19], meat substitute products offer a beneficial protein source while simultaneously reducing the consumption of saturated fat and cholesterol in comparison to meat. Advocates of traditional meat, thus, started lobbying in the USA, Australia, and the European Union to ban manufacturers of plant-based products from using any words associated with meat [20]. Due to the lack of standards for plant-based products, it is important to know what is available in supermarkets and what nutritional quality they offer compared with meat substitutes in other countries. Besides the nutritional quality, the price of a product plays a crucial role in consumers’ decision-making. Even though the expenditure elasticity for meat alternatives is still lower than that of conventional meat, consumers show a high willingness to purchase when plant-based meat substitutes are promoted [21]. This could indicate that the perceived price premium that one has to spend on meat substitutes is a weighing factor for the demand for meat substitutes.

To our current understanding, there is no existing study that investigates these aspects for the Austrian market. The situation in Austrian supermarkets is analyzed through standardized observations and secondary data analyses to obtain this information. The study builds on the design of Curtain and Grafenauer [22], who assessed the situation of plant-based meat alternatives in Australian supermarkets and focused on the ingredients and nutritional claims of plant-based meat alternatives. As price is an important factor in consumption decisions [23,24], the price was also implemented in this study’s design. A comparison with Australia is relevant to assess the market and draw parallels to a significantly larger and more developed market compared with the Austrian one [25,26]. These two countries share similar economic developments and above-average meat consumption in global comparison but are trending towards (AUS) or have already reached (AUT) “peak meat” [8,27]. Apart from these similarities, Austria and Australia exhibit different market forces. For example, Australia currently has the third fastest-growing market for vegan products in the world, following the United Arab Emirates and China [22,26]. The variation in market forces, along with their significant geographic separation, emphasizes the scientific importance of analyzing individual responses to the same global trend. The aim of this paper is, therefore, to answer the following questions:

(1) How do plant-based meat substitutes differ from conventional meat offered in Austrian supermarkets in terms of price, ingredients, and nutritional information?
(2) How do the results compare against products from Australia?

## 2. Materials and Methods

Standardized observations were conducted to collect data in May 2022 in Vienna, the capital city of Austria. The observation sites were nationwide supermarket chains (i.e., Billa, Billa Plus, Spar/Eurospar/Interspar, Hofer, and Lidl). These chains accounted for more than 90% of the market share in the Austrian food trade [28] and were chosen to reflect the range of products available to the majority of shoppers. To avoid any sold-out products, we visited several branches of each supermarket chain with different geographical locations and collected the prices, ingredients, and nutritional information of all plant-based meat alternatives that imitate minced meat or sausages. In order to guarantee the comparability of the results, the categories of minced meat and sausages are used because they are currently the most common forms of processed and plant-based meat substitutes available. For this paper, the category ‘minced meat’ includes all products labeled ‘minced meat’ as well as products based on minced meat, such as ‘burger patties’ or ‘meatballs’. For this work, sausage goods mean all types of sausages and sausage slices, including cold cuts and spreadable sausages, but not ham or bacon. Only plant-based products that imitate conventional minced meat or sausages are considered in this work. Other plant-based products that imitate meat or fish products or those that do not seek to emulate a specific meat or fish product, such as tofu or falafel, are not considered.

**Figure 1:**
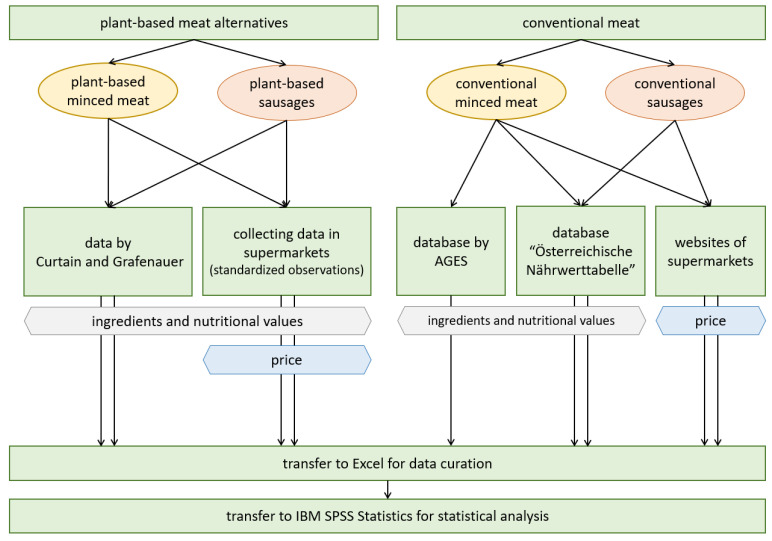
Flow chart of the research design.

The observation took place with the help of the Sortly inventory app. We collected data on the price and mass, the ingredients that could be attributed to a nutritional value, as well as the nutritional values of energy, fat (including saturated fatty acids), carbohydrates (including sugar), protein, and salt for each product. The data on ingredients and nutritional values were taken from the packaging of the individual products, in case of meat substitutes, or, in case of conventional meat, from databases of AGES [29] and the “Österreichische Nährwerttabelle”, which is published by dato Denkwerkzeuge in cooperation with the Institute of Nutritional Sciences of the University of Vienna [30]. The data was then processed using MS Excel and IBM SPSS Statistics (as can be seen in Figure 1).

The article by Curtain and Grafenauer [22] serves as an important foundation for our work, particularly with respect to the methods employed and statistical data analysis, which we closely followed to make our and their data comparable. Similar to our study, Curtain and Grafenauer [22] used photos to record data, including ingredients and nutritional information. The products were then classified into several categories, including ‘mince’ and ‘sausages’.

The datasets which were used and compared with data collected for this paper came from different sources depending on the intention of the comparison. For the comparison with Australian market data, we drew on the data published by Curtain and Grafenauer [22]. For the comparison of nutritional values of meat alternatives with Austrian data on conventional sausages, we drew on the databases of AGES [29] and the “Österreichische Nährwerttabelle” [30]. Data records on conventional minced meat come from the latter data set alone [30]. For price comparisons, products for the minced meat category and products for the sausage category were collected via the websites of Billa, Spar, and Hofer [22].

To ensure the comparability of the study, we used the same methods as Curtain and Grafenauer [22]. In the first step, the arithmetic mean, the standard deviation, the median, and the general distribution range of the data were computed. These statistical ratios are intended to screen the data and to make it comparable and analyzable. In the second step, we performed a Shapiro–Wilk test for normal distribution in the individual categories of nutritional values and price. This offers high test strength in contrast to other tests that test for normal distribution and is particularly suitable for testing smaller samples [31]. In the third step, we compared the mean values of the respective categories of the matching products (e.g., protein content in plant-based minced meat vs. conventional minced meat). If the normal distribution was present, this was done using independent sample *t*-tests for parametric data; if normal distribution could not be detected, the Mann–Whitney U test for non-parametric data was applied. These tests were used to compare the nutritional values and price of meat with the nutritional values and price of plant-based meat alternatives, as well as the nutritional values of Austrian plant-based meat alternatives with Australian ones.

## 3. Results

A total of 74 meat substitutes were evaluated, 64 of which are vegan. This corresponds to 87% of all products included. The remaining ten products are classified as vegetarian only. It is particularly striking that all products imitating minced meat are produced vegan, while of the products imitating sausages, just over 70% are produced vegan (Table 1). With 60 products, Australia offers one-third more products imitating minced meat than Austria, with 38. Quite contrarily, in the area of sausage products, one-quarter more options can be found in Austrian supermarkets, with 36 products compared with 29 in Australian supermarkets. Of the 36 imitation sausages, 8 products contain milk or egg protein. These were included in the analysis anyway, as they are at least one-third plant-based and marketed as meat alternatives. More detailed explanations of the ingredients follow in Section 3.2.

The Shapiro–Wilk test for normal distribution of the data shows that only the data in the categories price per kilogram, energy, and protein are normally distributed. Mean values of categories underlying normal distribution were tested using independent sample *t*-tests. If the normal distribution was not found, the Mann–Whitney U tests for data in the respective categories were conducted.

### 3.1. Price Comparison of Plant-Based Meat Alternatives to Conventional Meat in Austria

As an introduction to the price comparison, it is of interest to note the price/quantity ratio in which the product groups are offered in Austria. As shown in Table 2, at first glance in the supermarket, it appears as if plant-based meat alternatives are, on average, even slightly cheaper than conventional ones. However, when looking at the quantities that a pack contains, it is noticeable that plant-based meat alternatives are sold with significantly less content.

In order to compare the products studied, therefore, the price per kilogram was used. All prices per kilogram were recalculated to avoid any incorrect price tags in the supermarket (and indeed, nine of the seventy-four products displayed inaccurate prices per kilogram on the price tags in the supermarket). The average of the calculated prices per kilogram of the different product groups shows that plant-based meat alternatives are more expensive (see Table 3).

Plant-based minced meat is sold on average for almost five euros per kilogram more than conventional minced meat, which means that a buyer pays on average more for the same quantity. Plant-based sausages are, on average, about EUR 4.50 more expensive than conventional sausages, which corresponds to a price premium of almost 30%. In the case of minced meat, the price difference between conventional products to alternatives is higher in relative terms than for sausage products, although the absolute price per kilogram of minced meat is set lower than that of sausage products.

Furthermore, the standard deviations (Table 3) of the different product groups indicate a similar absolute price gap for both conventional minced meat and plant-based minced meat. In relative terms, this means a standard deviation of 22% from the mean for plant-based mince and a deviation of around 28% for conventional mince. The prices per kilogram of products belonging to the category of plant-based mince are thus somewhat closer to each other than those of conventional mince. In comparison, it is clear that there is a greater difference for sausages. At first glance, it is noticeable that conventional sausage products have a higher standard deviation than plant-based sausage products with a lower mean value. This means that plant-based sausages have a standard deviation of 24%, while conventional sausages come with a standard deviation of almost 43%. It is clearly visible that products in the plant-based sausage category have more similar prices per kilogram than conventional sausages, which have large differences in prices per kilogram.

As the median (Table 3) is more robust against outliers compared with the arithmetic mean, it may be suggested to give less influence on the overall result to any individual products that deviate very much from the mean. However, a direct comparison of median and mean values show that they are very close to each other, and thus, it can be assumed that across all analyzed product groups, there are only a few products that are far below or above the average price per kilogram.

Looking at the lowest and highest prices per kilogram per product group (Table 3), it is noticeable that the minima of the four product groups are more homogeneous than the maxima. The smallest prices per kilogram for conventional minced meat, plant-based sausages, and conventional sausages account for about 60% of the respective average price per kilogram. Only the minimum price per kilogram of plant-based minced meat is close to 50% of the average price per kilogram. The maximum prices per kilogram per product group, on the other hand, are not as equal. The highest prices per kilogram for alternative and conventional minced meat are around 136% and 133% of the mean price per kilogram. For alternative sausages, the maximum price per kilogram is 142% of the median price per kilogram, and the maximum price per kilogram for conventional sausages is 209%, which is about twice the median price per kilogram, far above the other maximum prices per kilogram.

**Figure 2:**
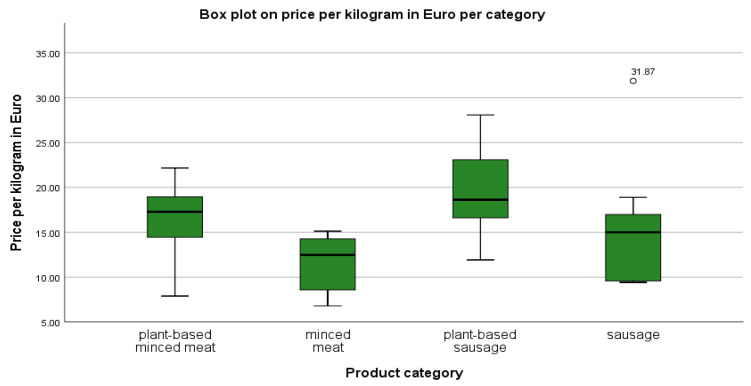
Box plot on price per kilogram in euros per category.

Figure 2 serves to make these differences graphically visible. It can be seen that the spectrum in which the prices lie is broader for plant-based products than for conventional meat products. It is particularly noticeable that one product in the conventional sausage category has a much higher price per kilogram (EUR 31.87) than other products in the same category.

In summary, plant-based minced meat (M = 16.32, Mdn = 17.28) costs, on average, 43% more per kilogram than conventional minced meat (M = 11.40, Mdn = 12.47), U = 55,000, z = −3.429, *p* = 0.001, and r = −0.495. Plant-based sausages (M = 19.72, Mdn = 18.63) cost on average 29% more per kilogram than conventional sausages (M = 15.24, Mdn = 15.00), U = 84.000, z = −2.487, *p* = 0.013, and r = −0.371. To put it simply, the plant-based meat alternatives analyzed in the course of this work are about a third more expensive and sold in smaller quantities than the equivalent conventional meat products. The prices of plant-based minced meat are more similar to conventional minced meat than the prices of plant-based sausage products are to conventional sausage products in almost all points examined (arithmetic mean ($\bar{x}$), standard deviation (σ), median, and range).

### 3.2. Ingredients of Plant-Based Meat Alternatives in Austria and Australia

This subsection identifies and evaluates the key ingredients of plant-based meat products in Austrian supermarkets and then examines them for differences and similarities with products available in Australia.

Table 4 and Table 5 show the listed ingredients of all included products in each category that cover the majority of a particular nutrient for that product. Some products list more than one ingredient that contributes to a particular nutrient. For example, a product may contain both wheat and soy protein. It is important to note that some ingredients contribute to more than one nutrient. These have been sorted into the nutrient group to which they contribute the most.

Table 4 shows that 55% of the products available in Austria in the plant-based mince category use pea protein as a source of protein. Of the 21 products that list pea protein, 13 also contain pea flour. For almost 30% of the products, soy protein is used as a protein base, and 21% of the products contain, among others, wheat protein as a source of protein. For the products sold in Austria in the category of plant-based minced meat, the ingredients sunflower oil, coconut oil/fat, and rapeseed oil are mainly relevant to fat supply. Almost 90% of the products contain at least one of these three ingredients. A total of 53% of the product group of plant-based minced meat explicitly mention the word “sugar” among the ingredients. Potato starch is the most frequently found carbohydrate supplier in the products, at 45%.

Table 5 shows that—as in the case of plant-based minced meat—pea protein is the most frequently used protein source in plant-based sausage products, accounting for 56%. Wheat protein is named as a protein source in 36% of the products, and soy protein in 19%. What was particularly striking about plant-based sausage products compared with plant-based mince was that there are also products that make use of both vegan and vegetarian protein, although the product itself is still plant-based. Overall, 10 of the 36 products (28%) contained either chicken egg and/or milk protein in addition to a plant-based protein source, almost exclusively mixed with pea protein. For plant-based sausages, all products use at least one of the following three ingredients: sunflower oil, rapeseed oil, and/or coconut oil/fat as a fat source. A total of 64% of the plant-based sausage product group explicitly mention the word “sugar” among the ingredients. Compared with plant-based minced meat, potato starch is found in significantly fewer products in plant-based sausage products (28%). On the other hand, 6 of the 36 products (17%) contain rice flour, which is not mentioned once in plant-based minced meat.

Compared with Australia, it is striking that in the study by Curtain and Grafenauer [22], only soy protein, pea protein, soybeans, hydrolyzed vegetable protein, mycoprotein, and almonds were recorded, but no wheat protein-based products. This stands in contrast to the ingredients of Austrian products. What unites products from both countries is that certain products are made using soy or pea protein. In the nutritional category of fat, it appears that products available in Australia contain a wider range of ingredients. In Australian products, the nutritional category carbohydrates include the ingredient tapioca, which is not present in any of the included products in Austria.

In summary, the most commonly used protein source for plant-based minced meat and sausage products in Austria is pea protein, accounting for 55% and 56%, respectively. The second most common protein source is soy protein for plant-based minced meat at close to 30% and wheat protein for plant-based sausages at 36%. In Australia, pea and soy proteins are also frequently used, but unlike in Austria, wheat protein is not utilized. Of the 48 ingredients evaluated, 15 ingredients can be found in both Austrian and Australian products. However, the remaining 33 ingredients can only be found in products that are available in either Austria or Australia. This means that—quite surprisingly—only 31% of the listed ingredients are found in products in both countries.

### 3.3. Comparison of Nutritional Values of Plant-Based Meat Alternatives to Conventional Meat

In order to be able to assess how well plant-based meat alternatives imitate their meat equivalents in Austria with regard to nutritional values, a comparison is made. However, before we turn to compare meat substitutes and conventional meat, we present a comparison of the two plant-based product classes in our study: plant-based minced meat and plant-based sausages. Table 6 provides an overview of the most important statistical key figures for the nutritional values of energy, fat (including saturated fatty acids), carbohydrates (including sugar), protein, and salt for plant-based minced meat and plant-based sausages. It can be seen that plant-based minced meat has higher mean values than plant-based sausage products in the nutritional categories of energy, fat (including saturated fatty acids), carbohydrates, and protein. Conversely, plant-based sausage products have higher mean values in the nutritional categories of fat and salt. Remarkably, the mean value, the standard deviation, and the minimum in the category sugar are similar for both plant-based meat alternatives considered, but not the median and the maximum value. At second glance, it becomes apparent that there are often large ranges behind the mean values, which indicates that the individual products consist of different ingredients. A look at the standard deviation also helps to assess whether the products in the individual nutritional categories have rather homogeneous or heterogeneous compositions.

Through independent sample *t*-tests, a comparison of plant-based minced meat with conventional minced meat was conducted and shows that at a confidence interval of 95%, four out of seven nutritional groups considered have a non-significant result, implying similar means at 57% of the nutritional values and a significant difference at 43% of the nutritional means. At the same confidence interval, two of the seven nutritional groups considered show a non-significant result in *t*-tests comparing plant-based sausages with conventional sausages, implying similar means in 29% of the nutritional values and a significant difference in 71% of the mean nutritional values.

Table 7 shows the results of the independent sample *t*-test for the nutritional values of plant-based and conventional minced meat. At a confidence interval of 95%, plant-based minced meat significantly differs from conventional minced meat in the nutritional categories of carbohydrates (including sugar) and salt. Plant-based mince has higher values in all three categories just mentioned. The nutritional categories energy, fat (including saturated fatty acids), and protein show no significant difference. Plant-based minced meat, therefore, has a similar content of energy, fat (including saturated fatty acids), and protein as conventional minced meat.

The results of the independent sample *t*-test regarding the nutritional values of plant-based and conventional sausage indicate that at a significance level of 5% (confidence interval 95%), there is a significant difference of means in the nutritional value categories energy, fat (including saturated fatty acids), and carbohydrates (including sugar). In the categories of energy and fat (including saturated fatty acids), conventional sausage products have higher values, while the opposite is true for carbohydrates (including sugar), where plant-based sausage products score comparatively higher. The categories protein and salt show non-significant results. Plant-based sausage products have a similar protein and salt content as conventional sausage products.

In conclusion, our results show that plant-based meat substitutes are across groups and nutritional values superior to conventional meat since they contain less fat and are equal in their amount of protein. Plant-based sausages especially perform significantly better than their conventional counterpart in terms of energy (less is more), fat (including saturated fatty acids), and salt. Only carbohydrates (including sugar) perform worse in both plant-based mince and plant-based sausages.

### 3.4. Nutritional Values of Plant-Based Meat Alternatives in Austria and Australia

A comparative analysis is conducted to evaluate the differences in nutritional values among plant-based meat alternatives in Austria and Australia. At a confidence interval of 95%, the results of the independent sample *t*-tests in Table 8 show significant results in each of the seven nutritional categories for the nutritional values of plant-based minced meat and significant results in six out of seven nutritional categories for the nutritional values of plant-based sausages in Austrian and Australian supermarkets.

For plant-based minced meat, the mean values of energy, fat (including saturated fatty acids), carbohydrates (including sugar), protein, and salt are significantly different, and none of the nutritional categories show similarities between Austrian and Australian plant-based mince. Plant-based minced meat available in Austria shows higher mean values for the nutritional values of energy, fat (including saturated fatty acids), protein, and salt. For the nutritional values of carbohydrates (including sugar), the equivalent products available in Australia have higher mean values.

There are significant differences in the nutritional categories of energy, fat (including saturated fatty acids), carbohydrates (including sugar), and salt. Similar to the comparison of mean values of Austrian and Australian plant-based minced meat, the results also show that plant-based sausage products available in Austria have higher mean values in the nutritional categories energy, fat (including saturated fatty acids), and salt, while products available in Australia have higher values in the nutritional category carbohydrates (including sugar). Only the *t*-test of the mean values of proteins does not produce a significant result. With a 95% probability that a value lies within the confidence interval, this is the highest result of all independent sample *t*-tests conducted in this work and shows the close similarity in terms of protein contained.

In summary, it can be said that Austrian plant-based minced meat could not show a similar mean value in any nutritional category compared with Australian plant-based minced meat. Compared with Austrian plant-based sausage, Australian plant-based sausage only has a similar mean value in the nutritional category of protein. It can be shown that plant-based products on the respective markets are highly different in terms of nutritional value and should therefore be viewed as completely different products depending on the country.

## 4. Discussion

One of the most noteworthy outcomes of our study was the finding that plant-based meat substitutes (both minced meat and sausages) in Austrian supermarkets (and, to a lesser extent, in Australia) exhibit protein contents similar to that of conventional meat. Our results highlight that plant-based meat alternatives may indeed serve as important sources of “alternative proteins”.

As plant-based meat alternatives in Austria are typically priced between one-third to almost twice that of traditional meat, it prompts the question: “What is the cost of achieving optimal nutritional levels?” Because the processing of the energy comparison within this work showed that the mean values of plant-based and conventional minced meat in Austria do not differ significantly from each other, the answer to the question posed is only relevant for the category of plant-based sausage products in comparison to conventional sausage products because these show a significant difference in the mean energy value. Plant-based meat alternatives are not suitable for saving money, as the price per kilogram comparison has already shown. Since plant-based products tend to have lower energy values than the equivalent meat product for the same quantity, it can be roughly estimated that at a price per energy unit comparison (kJ), the plant-based products turn out to be more expensive on average than the equivalent meat product. However, given the high prevalence of overweight and obese individuals aged 18 and over in Austria (51%) [32] and Australia (67%) [33], it can be considered positive that plant-based meat substitutes offer commensurate protein content while providing lower caloric intake than conventional meat.

When comparing the ingredients, it was noticeable that hydrolyzed vegetable protein (HDV) and mycoprotein, which are popular in Australia, are not used in Austria. Both are commonly used in other European countries tough [34,35,36]. Because the evaluated nutritional values of the plant-based meat alternatives in Austria considered in this paper showed more similar mean values to meat available in Austria than to Australian plant-based meat alternatives, the question arises whether plant-based meat alternatives available in Australia also have more similar mean nutritional values to meat available in Australia, or whether these imitate meat worse in terms of nutritional values than the products sold in Austria. After analyzing the independent sample *t*-tests conducted by Curtain and Grafenauer [22] (for Australia), five out of twenty-one *t*-tests showed similar mean values looking at plant-based and conventional meat, which equals 24%. In comparison: Austrian data shows 43% similar nutrient means across the same nutrient categories. Comparing similar means for Australian plant-based to conventional meat (24%) with Australian plant-based to Austrian plant-based meat (7%) shows that plant-based meat alternatives sold in Australia are more similar to conventional meat available in Australia than to plant-based meat alternatives available in Austria (in terms of nutritional value). This highlights the difference in markets and food standards between the EU (which has the highest standards internationally) and Australia [37,38] and indicates the necessity for an internal EU comparison in further studies. We used the most comprehensive data available for the Australian market (i.e., Curtain and Grafenauer, 2019 [22]). However, they are three years old, arguably a long time, and the market might have changed. Therefore, we encourage future research to make longitudinal studies to better assess the development of the meat-alternative markets.

Similar studies also found different nutritional compositions of plant-based meat alternatives to conventional meat in Norway [39], Sweden [40], the UK [41], Brazil [42], and the USA [43,44]. A Swedish report by Bryngelsson et al. [45] concluded that plant-based mince, fillet, as well as nuggets, would be the healthier alternative after considering the nutritional composition compared with conventional meat, although future work should address the biological availability of iron, the quality of protein and the impact of product manufacturing. The findings of Ložnjak Švarc et al. [35] from Denmark show a wide range in the nutrient composition of plant-based meat alternatives and therefore recommend nutritional guidance when replacing animal products with plant-based alternatives. In accordance with these findings, an analysis of largely North American plant-based products by Bohrer [16] indicates that consumers should investigate the nutritional differences between plant-based and conventional meat products. Adding on, a study by Salomé et al. [46] in France shows that the right choice of ingredients can lead to a nutritionally valuable meat substitute. Consumers, though, perceive the nutritional value of meat substitutes very differently, a current study shows [47]. While some groups expect positive health effects, others are generally very skeptical of the products. A systematic literature review by Zahari et al. [48] concluded that the technical processes of manufacturing and researching plant-based meat alternatives are still in their infancy. Processing of proteins into a meat-like texture, including their ability to be absorbed by the human body, as well as the general nutritional composition of the products, still has potential for improvement in the future [48]. Van Vliet et al. [49] found that even with similar nutritional values, plant-based proteins get absorbed differently by the human body, which is consistent with findings from Mayer Labba and colleagues [40].

To sum up the discussion: it can be said that—for the Austrian market—the price of meat substitutes and conventional meat significantly differ from each other, the same as the nutritional value of these products. Studies from other countries that also compared the nutritional value of plant-based meat alternatives with meat came to similar results: The nutritional composition of meat substitutes is largely different from conventional meat [22,40,43]. Contrary to our initial belief also, the ingredients of meat substitutes significantly differ between products on the Austrian and products on the Australian market.

## 5. Conclusions

Reflecting on our initial research question, we set out to study the market supply and nutritional values for plant-based meat substitutes in Austria and compare them to the Australian one. It can be concluded that plant-based meat alternatives offer comparable protein content to conventional meat but significantly differ in other nutritional aspects. Furthermore, the nutrient profile of plant-based meat products in the Australian market also exhibits differences compared with the studied alternatives. This points out the differences in food standards and market supply in geographically distant yet culturally similar regions. Additionally, it is striking to see that plant-based products are still sold with a significant price premium compared to conventional meat, even though they can be accounted as equal, especially when looking for an alternative source of protein, which also contains fewer calories and fat (including saturated fatty acids). Therefore, the consumption of plant-based meat alternatives appears to have a significantly positive impact on the individual as well as global health (when looking at emissions produced during meat production) and should be an incentive for policymakers to reduce the price of meat alternatives, for instance, through subsidies. In conclusion, it can be said that the market for meat substitutes is a steadily developing one that needs to be promoted if one has both human health and the well-being of the planet in mind.

Furthermore, it is important to point out the limitations of this work and to reflect on the results critically. It is particularly relevant to mention that the samples compared have different sizes. The data of the standardized observation reflect the most complete picture possible of the Austrian supermarkets, although there may be differences in the geographical coverage. The sample was taken from supermarkets in Vienna, as it can be assumed that they offer the greatest variety of products across Austria. Smaller exemplary samples were taken for both the price comparison data and the nutritional data of meat available in Austria, as the inventory of the comprehensive meat supply in Austrian supermarkets did not correspond to the central research interest. Nevertheless, it must be noted that this can lead to a distorted basis of comparison of conventional meat products—as can be seen, for example, in the category “prices of sausages”.

As only data from products imitating minced meat (including burger patties and meatballs) or sausages were included in this study, it would make sense to further analyze other plant-based imitations, such as nuggets, schnitzel, steak, fillet, fish fingers, shrimps or salmon, to provide a complete picture of the market supply of plant-based meat alternatives in Austria and paint a comprehensive picture over time. This scientific article contributes to the existing body of knowledge by providing valuable insights into the market trends, nutritional values, and pricing dynamics of plant-based meat substitutes. As they are contributing to informed consumer purchasing decisions, future research should include how the price ratio of plant-based meat alternatives will develop, whether the ingredients will change over time and align globally, or whether the average nutritional values in respective countries will be more homogeneous to conventional meat or other plant-based meat alternatives in the future. For consumers, an independent evaluation of the individual products, for instance, on the degree of processing and naturalness in meat analogs, would be helpful, given the so-called “clean label” trend [50]. This evaluation would provide easily understandable information to support consumer acceptance and purchasing decisions. For governmental organizations or policymakers, these insights offer a stepping stone for informed decision-making regarding the promotion of plant-based diets, the reduction of obesity rates, and the formulation of regulatory frameworks. For industry stakeholders, this study holds valid information on how to penetrate and develop the Austrian market for meat alternatives even deeper. New avenues for future research should be explored, such as investigating the underlying factors influencing pricing differentials and exploring strategies to enhance the affordability and accessibility of plant-based alternatives. This could foster a healthier diet, not only on the individual but also on the environmental scale.

To conclude, more research on markets for meat substitutes is needed as they are dynamically developing and are relevant to global development from economic, environmental, and social perspectives. We hope that our research will contribute to the knowledge base in this field and serve as an inspiration and starting point for future country comparisons.

## Figures and Tables

**Table 1:** Product overview Austria vs. Australia.

Plant-Based Meat Substitutes	Number of Products in Austria	Thereof Vegan	Number of Products in Australia ^1^
Minced meat	38	38 ≙ 100%	60
Sausages	36	26 ≙ 72%	29
**Sum**	**74**	**64** ** ≙ 87%**	**89**

^1^ Data from Curtain and Grafenauer 2019 [22].

**Table 2:** Price in euros and quantity in grams per pack for each product group.

Product Group	Mean Price (EUR)	Mean Quantity (g)
Plant-based minced meat	3.51	229
Conventional minced meat	4.60	436
Plant-based sausages	2.68	143
Conventional sausages	3.47	282

**Table 3:** Descriptive indicators for price per kilogram (kg) for each product group in euro (EUR).

Product Group	Mean	Standard Dev.	Median	Min.	Max.
Plant-based minced meat	16.32	±3.66	17.28	7.90	22.17
Conventional minced meat	11.40	±3.16	12.47	6.79	15.12
Plant-based sausages	19.72	±4.76	18.63	11.92	28.06
Conventional sausages	15.24	±6.54	15.00	9.42	31.87

**Table 4:** Ingredients in plant-based minced meat that contribute to nutritional values (by frequency).

Nutritional Value	Listed Ingredients (Product Number n of 38)
Protein	Pea protein (21), pea flour (14), soy protein (11), wheat protein (8), soy flour (6), yeast extract (5), rice protein (3), soybeans (2), lentils (1), oat protein (1), and sunflower protein (1)
Fat	Sunflower oil (23), coconut oil/fat (17), rapeseed oil (16), cocoa butter (1), and walnuts (1)
Carbohydrates	(Caramelized) sugar (20), potato starch (17), barley malt extract (10), tomato paste (6), maize starch (5), wholemeal oat flour (5), wheat flour (5), potato (3), maize flour (2), chickpea flour (1), wheat starch (1), spelt (1), and kidney beans (1)

**Table 5:** Ingredients in plant-based sausages that contribute to nutritional values (by frequency).

Nutritional Value	Listed Ingredients (Product Number n of 36)
Protein	Pea protein (20), wheat protein (13), chicken egg protein (10), milk protein (8), soy protein (7), pea flour (5), yeast extract (5), potato protein (2), sunflower protein (2), rice protein (1), soybeans (1), oat protein (1), and field bean protein (1)
Fat	Sunflower oil (20), rapeseed oil (16), coconut oil/fat (14), and palm oil/fat (2)
Carbohydrates	(Caramelised) sugar (23), potato starch (10), rice (flour) (6), wheat flour (3), potato (2), wheat starch (2), corn starch (1), and tomato paste (1)

**Table 6:** Nutritional values per 100 g of plant-based minced meat and sausages (arithmetic mean ($\bar{x}$), standard deviation (σ), and median with range).

	Plant-Based Minced Meat		Plant-Based Sausages	
Nutritional Value	$\bar{x}$ ± σ	Median (Min–Max)	$\bar{x}$ ± σ	Median (Min–Max)
Energy (kJ)	902 ± 179	955 (529–1166)	865 ± 218	861 (348–1356)
Fat (g)	12.5 ± 5.6	14.0 (0.5–19.0)	14.3 ± 4.1	15.5 (4.9–24.0)
Thereof saturated fatty acids (g)	4.8 ± 4.9	1.9 (0.1–16.0)	3.9 ± 3.3	1.5 (0.6–11.0)
Carbohydrates (g)	7.6 ± 4.9	6.0 (2.0–22.0)	5.2 ± 2.5	4.8 (2.9–15.0)
Thereof sugar (g)	1.3 ± 0.8	1.0 (0.0–2.8)	1.3 ± 0.8	1.1 (0.0–3.5)
Protein (g)	15.9 ± 4.6	16.0 (7.8–27.0)	13.3 ± 7.6	10.5 (2.5–31.0)
Salt (g)	1.5 ± 0.4	1.5 (0.7–2.6)	2.1 ± 0.7	1.9 (1.1–3.3)

**Table 7:** Nutritional values per 100 g of plant-based and conventional minced meat and sausage (arithmetic mean ($\bar{x}$), standard deviation (σ), and *p*-value of the independent sample *t*-test).

	Plant-Based Minced Meat	Conventional Minced Meat		Plant-Based Sausages	Conventional Sausages	
Nutritional Value	$\bar{x}$ ± σ	$\bar{x}$ ± σ	*p*-Value	$\bar{x}$ ± σ	$\bar{x}$ ± σ	*p*-Value
Energy (kJ)	902 ± 179	990 ± 109	0.345	865 ± 218	1364 ± 244	<0.001 ***
Fat (g)	12.5 ± 5.6	17.7 ± 3.1	0.081 *	14.3 ± 4.1	29.0 ± 4.6	<0.001 ***
Thereof saturated fatty acids (g)	4.8 ± 4.9	7.4 ± 1.4	0.295	3.9 ± 3.3	11.7 ± 2.1	<0.001 ***
Carbohydrates (g)	7.6 ± 4.9	0.1 ± 0.1	0.005 ***	5.2 ± 2.5	0.6 ± 0.4	<0.001 ***
Thereof sugar (g)	1.3 ± 0.8	0.0 ± 0.0	0.005 ***	1.3 ± 0.8	0.5 ± 0.3	0.011 **
Protein (g)	15.9 ± 4.6	19.1 ± 1.1	0.182	13.3 ± 7.6	15.5 ± 3.5	0.490
Salt (g)	1.5 ± 0.4	0.2 ± 0.0	<0.001 ***	2.1 ± 0.7	2.7 ± 0.8	0.060 *

* = significant at the 10% level; ** = significant at the 5% level; and *** = significant at the 1% level.

**Table 8:** Nutritional values per 100 g of plant-based minced meat in Austria and Australia (arithmetic mean ($\bar{x}$), standard deviation (σ), and *p*-value of the independent sample *t*-test).

	Plant-Based Minced Meat (Austria, n = 38)	Plant-Based Minced Meat ^1^ (Australia, n = 60)		Plant-Based Sausage (Austria, n = 36)	Plant-Based Sausage ^1^ (Australia, n = 29)	
Nutritional Value	$\bar{x}$ ± σ	$\bar{x}$ ± σ	*p*-Value	$\bar{x}$ ± σ	$\bar{x}$ ± σ	*p*-Value
Energy (kJ)	902 ± 179	655 ± 216	<0.001 ***	865 ± 218	735 ± 155	0.007 ***
Fat (g)	12.5 ± 5.6	6.3 ± 5.0	<0.001 ***	14.3 ± 4.1	7.9 ± 3.8	<0.001 ***
Thereof saturated fatty acids (g)	4.8 ± 4.9	1.8 ± 2.4	<0.001 ***	3.9 ± 3.3	2.4 ± 2.2	0.032 **
Carbohydrates (g)	7.6 ± 4.9	12.3 ± 7.3	<0.001 ***	5.2 ± 2.5	11.4 ± 6.2	<0.001 ***
Thereof sugar (g)	1.3 ± 0.8	2.7 ± 2.4	<0.001 ***	1.3 ± 0.8	2.2 ± 1.9	0.020 **
Protein (g)	15.9 ± 4.6	11.7 ± 5.6	<0.001 ***	13.3 ± 7.6	13.4 ± 6.0	0.953
Salt (g)	1.5 ± 0.4	1.0 ± 0.6	<0.001 ***	1.5 ± 0.4	1.0 ± 0.6	<0.001 ***

** = significant at the 5% level and *** = significant at the 1% level. ^1^ Data from Curtain and Grafenauer [22].

## Data Availability

The data supporting the findings of this study are available from the corresponding author upon reasonable request.

## References

[1] Pachauri R.K., Meyer L.A., IPCC (2014). Climate Change 2014: Synthesis Report. Contribution of Working Groups I, II and III to the Fifth Assessment Report of the Intergovernmental Panel on Climate Change.

[2] Europäische Kommission Übereinkommen von Paris. https://ec.europa.eu/clima/eu-action/international-action-climate-change/climate-negotiations/paris-agreement_de.

[3] Poore J., Nemecek T. (2018). Reducing Food’s Environmental Impacts through Producers and Consumers. Science.

[4] Xu X., Sharma P., Shu S., Lin T.-S., Ciais P., Tubiello F.N., Smith P., Campbell N., Jain A.K. (2021). Global Greenhouse Gas Emissions from Animal-Based Foods Are Twice Those of Plant-Based Foods. Nat. Food.

[5] Aiking H., de Boer J. (2020). The next Protein Transition. Trends Food Sci. Technol..

[6] Ritchie H., Roser M. Land Use. https://ourworldindata.org/land-use.

[7] Food and Agriculture Organization of the United Nations (2022). Food Outlook—Biannual Report on Global Food Markets.

[8] Whitton C., Bogueva D., Marinova D., Phillips C.J.C. (2021). Are We Approaching Peak Meat Consumption? Analysis of Meat Consumption from 2000 to 2019 in 35 Countries and Its Relationship to Gross Domestic Product. Animals.

[9] Barnard N., Levin S., Trapp C. (2014). Meat Consumption as a Risk Factor for Type 2 Diabetes. Nutrients.

[10] Godfray H.C.J., Aveyard P., Garnett T., Hall J.W., Key T.J., Lorimer J., Pierrehumbert R.T., Scarborough P., Springmann M., Jebb S.A. (2018). Meat Consumption, Health, and the Environment. Science.

[11] Tilman D., Clark M. (2014). Global Diets Link Environmental Sustainability and Human Health. Nature.

[12] Statistik Austria, AMA-Marketing Entwicklung Des Pro-Kopf-Verbrauches von Fleisch Inkl. Geflügel Gesamt in Österreich (in Kg). https://amainfo.at/fileadmin/user_upload/Pro_Kopf_Verbrauch_Fleisch.pdf.

[13] Sozialministerium 7 Stufen Zur Gesundheit. https://www.sozialministerium.at/Themen/Gesundheit/Lebensmittel-Ernaehrung/Ern%C3%A4hrungsempfehlungen/Ern%C3%A4hrungspyramide0.html.

[14] Onwezen M.C., Bouwman E.P., Reinders M.J., Dagevos H. (2021). A Systematic Review on Consumer Acceptance of Alternative Proteins: Pulses, Algae, Insects, Plant-Based Meat Alternatives, and Cultured Meat. Appetite.

[15] Aschemann-Witzel J., Gantriis R.F., Fraga P., Perez-Cueto F.J.A. (2021). Plant-Based Food and Protein Trend from a Business Perspective: Markets, Consumers, and the Challenges and Opportunities in the Future. Crit. Rev. Food Sci. Nutr..

[16] Bohrer B.M. (2019). An Investigation of the Formulation and Nutritional Composition of Modern Meat Analogue Products. Food Sci. Hum. Wellness.

[17] Tso R., Forde C.G. (2021). Unintended Consequences: Nutritional Impact and Potential Pitfalls of Switching from Animal- to Plant-Based Foods. Nutrients.

[18] Seehafer A., Bartels M. (2019). Meat 2.0—The Regulatory Environment of Plant-Based and Cultured Meat. Eur. Food Feed. Law Rev..

[19] Kumar P., Chatli M.K., Mehta N., Singh P., Malav O.P., Verma A.K. (2017). Meat Analogues: Health Promising Sustainable Meat Substitutes. Crit. Rev. Food Sci. Nutr..

[20] ProVeg Die Benennung Vegetarischer Und Veganer Lebensmittel. https://proveg.com/de/was-wir-tun/politische-arbeit/lebensmittelkennzeichnung/benennung-veggie-produkte/.

[21] Zhao S., Wang L., Hu W., Zheng Y. (2023). Meet the Meatless: Demand for New Generation Plant-based Meat Alternatives. Appl. Econ. Perspect. Policy.

[22] Curtain F., Grafenauer S. (2019). Plant-Based Meat Substitutes in the Flexitarian Age: An Audit of Products on Supermarket Shelves. Nutrients.

[23] Carlsson F., Kataria M., Lampi E. (2022). How Much Does It Take? Willingness to Switch to Meat Substitutes. Ecol. Econ..

[24] Clark L.F., Bogdan A.-M. (2019). The Role of Plant-Based Foods in Canadian Diets: A Survey Examining Food Choices, Motivations and Dietary Identity. J. Food Prod. Mark..

[25] Mascaraque M. (2020). Going Plant-Based: The Rise of Vegan and Vegetarian Food.

[26] Wan L. Fact Not Fad: Why the Vegan Market is Going from Strength-to-Strength in Australia. https://www.foodnavigator-asia.com/Article/2018/04/25/Fact-not-fad-Why-the-vegan-market-is-going-from-strength-to-strength-in-Australia.

[27] Statistik Austria Versorgungsbilanzen. https://www.statistik.at/statistiken/land-und-forstwirtschaft/landwirtschaftliche-bilanzen/versorgungsbilanzen.

[28] Statista Research Department Umsatz Der Führenden Unternehmen Im Lebensmitteleinzelhandel in Österreich 2020. https://de.statista.com/statistik/daten/studie/215847/umfrage/umsatz-der-groessten-haendler-in-oesterreich/#statisticContainer.

[29] AGES Lebensmittellupe. https://www.lebensmittellupe.at/index.php?id=1818.

[30] Dato Denkwerkzeuge Institut für Ernährungswissenschaften Universität Wien Österreichische Nährwerttabelle. https://oenwt.at/content/naehrwert-suche/.

[31] Field A. (2017). Discovering Statistics Using IBM SPSS Statistics.

[32] Statistik Austria Übergewicht Und Adipositas. https://www.statistik.at/statistiken/bevoelkerung-und-soziales/gesundheit/gesundheitsverhalten/uebergewicht-und-adipositas.

[33] Australian Institute of Health and Welfare Overweight and Obesity. https://www.aihw.gov.au/reports/australias-health/overweight-and-obesity.

[34] Dataintelo Hydrolyzed Vegetable Protein Market Research Report. https://dataintelo.com/report/hydrolyzed-vegetable-protein-market-report/.

[35] Ložnjak Švarc P., Jensen M.B., Langwagen M., Poulsen A., Trolle E., Jakobsen J. (2022). Nutrient Content in Plant-Based Protein Products Intended for Food Composition Databases. J. Food Compos. Anal..

[36] Methven L. (2012). Natural Food and Beverage Flavour Enhancer. Natural Food Additives, Ingredients and Flavourings.

[37] Bundesinstitut für Risikobewertung (BfR) Lebensmittel in Der EU Unterliegen Strengsten Standards. https://www.bfr.bund.de/de/presseinformation/2019/15/lebensmittel_in_der_eu_unterliegen_strengsten_standards-240646.html.

[38] Davis J. British, Australian Food Standard Differences Causing Angst in Free Trade Deal. https://www.abc.net.au/news/2021-06-11/british-australian-food-standard-differences-causing-angst/100205024.

[39] Tonheim L.E., Austad E., Torheim L.E., Henjum S. (2022). Plant-Based Meat and Dairy Substitutes on the Norwegian Market: Comparing Macronutrient Content in Substitutes with Equivalent Meat and Dairy Products. J. Nutr. Sci..

[40] Mayer Labba I.-C., Steinhausen H., Almius L., Bach Knudsen K.E., Sandberg A.-S. (2022). Nutritional Composition and Estimated Iron and Zinc Bioavailability of Meat Substitutes Available on the Swedish Market. Nutrients.

[41] Alessandrini R., Brown M.K., Pombo-Rodrigues S., Bhageerutty S., He F.J., MacGregor G.A. (2021). Nutritional Quality of Plant-Based Meat Products Available in the UK: A Cross-Sectional Survey. Nutrients.

[42] Penna Franca P.A., Duque-Estrada P., da Fonseca e Sá B.F., van der Goot A.J., Pierucci A.P.T.R. (2022). Meat Substitutes—Past, Present, and Future of Products Available in Brazil: Changes in the Nutritional Profile. Future Foods.

[43] Cole E., Goeler-Slough N., Cox A., Nolden A. (2022). Examination of the Nutritional Composition of Alternative Beef Burgers Available in the United States. Int. J. Food Sci. Nutr..

[44] Harnack L., Mork S., Valluri S., Weber C., Schmitz K., Stevenson J., Pettit J. (2021). Nutrient Composition of a Selection of Plant-Based Ground Beef Products Available in the United States. J. Acad. Nutr. Diet.

[45] Bryngelsson S., Moshtaghian H., Bianchi M., Hallström E. (2022). Nutritional Assessment of Plant-Based Meat Analogues on the Swedish Market. Int. J. Food Sci. Nutr..

[46] Salomé M., Mariotti F., Nicaud M.C., Dussiot A., Kesse-Guyot E., Maillard M.N., Huneau J.F., Fouillet H. (2022). The Potential Effects of Meat Substitution on Diet Quality Could Be High If Meat Substitutes Are Optimized for Nutritional Composition—A Modeling Study in French Adults (INCA3). Eur. J. Nutr..

[47] Garaus M., Garaus C. (2023). US Consumers’ Mental Associations with Meat Substitute Products. Front. Nutr..

[48] Zahari I., Östbring K., Purhagen J.K., Rayner M. (2022). Plant-Based Meat Analogues from Alternative Protein: A Systematic Literature Review. Foods.

[49] van Vliet S., Bain J.R., Muehlbauer M.J., Provenza F.D., Kronberg S.L., Pieper C.F., Huffman K.M. (2021). A Metabolomics Comparison of Plant-Based Meat and Grass-Fed Meat Indicates Large Nutritional Differences despite Comparable Nutrition Facts Panels. Sci. Rep..

[50] Delgado-Pando G., Ekonomou S.I., Stratakos A.C., Pintado T. (2021). Clean Label Alternatives in Meat Products. Foods.

